# Papillotubular Adenoma of The Ampullary Region

**DOI:** 10.1155/1989/92374

**Published:** 1989

**Authors:** Minoru Numata, Toru Noguchi

**Affiliations:** Department of Surgery Shinshu University School of Medicine 3-1-1 Asahi-cho Matsumoto 390 Japan

## Abstract

Papillotubular adenoma of the ampullary region in a rare tumor which has the potential for malignant transformation. Although reported more frequently nowadays, ampullary tumors are difficult to diagnose before operation unless they cause obstructive jaundice. Occasionally they are detected by ultrasonography, computed tomography or ERCP in patients who complain of nonspecific upper abdominal discomfort.

We report a case of a periampullary papilotubular adenoma causing generalized pruritus and weight loss but no jaundice. The tumor was extirpated by pancreaticoduodenectomy, and the patient has remained healthy for over five years.


*HPB Surgery* 1989, Vol. 1, pp. 345–351
Reprints available directly from the publisher
Photocopying permitted by license only

© 1989 Harwood Academic Publishers GmbH
Printed in Great Britain

### CASE REPORT
### PAPILLOTUBULAR ADENOMA OF THE AMPULLARY REGION

MINORU NUMATA and TORU NOGUCHI

*Department of Surgery, Shinshu University of Medicine*

*(Received 18 July, 1988)*

Papillotubular adenoma of the ampullary region in a rare tumor which has the potential for malignant transformation. Although reported more frequently nowadays, ampullary tumors are difficult to diagnose before operation unless they cause obstructive jaundice. Occasionally they are detected by ultrasonography, computed tomography or ERCP in patients who complain of nonspecific upper abdominal discomfort.

We report a case of a periampullary papilotubular adenoma causing generalized pruritus and weight loss but no jaundice. The tumor was extirpated by pancreaticoduodenectomy, and the patient has remained healthy for over five years.

KEY WORDS: Papillotubular adenoma, ampullary region, malignant transformation.

### INTRODUCTION

Tubular adenoma is a rare variant among benign tumors in the ampullary region[1]. Adenoma of the papilla of Vater can undergo malignant change[2], both mucocystic and the tubular types[3]. It is usually difficult to diagnose benign tumors of the ampulla at an early stage. Occasionally they cause obstructive jaundice, but in some patients with vague upper abdominal pain or pruritus they may be revealed by routine examinations such as ultrasonography, computed tomography or ERCP. We report a case of tubular adenoma of the ampullary region discovered during a course of such routine investigations.

### CASE REPORT

A 50-year-old man who had suffered from liver dysfunction for two years referred to our hospital for routine physical examination of the biliary tract because of generalized pruitus, weight loss (2 to 3 kg/month) and occasional pyrexia (39°C to 40°C). On physical examination, palmar erythaema and vascular spiders of the abdominal wall were observed. Laboratory data showed slight anemia and mildly deranged liver function tests. Random estimation of serum amylase and blood sugar were normal. Ultrasonography of the upper abdomen revealed dilatation of the intrahepatic ducts with the so called "parallel channel" or "shot gun" sign. A dilated common bile duct was demonstrated by ERCP examination, but no biopsy was performed at this time. Exploratory laparotomy was performed for suspicion of

Address correspondence to Dr. Numata, Department of Surgery, Shinshu University School of Medicine, 3-1-1 Asahi-cho, Matsumoto 390, Japan carcinoma of the pancreatic head, and the tumor was removed by pancreaticoduodenectomy. No lymph node involvement was observed in the surrounding tissues. The patient made an uneventful recovery and remained well and free of recurrence for 5 years.

### *Pathological findings*

Gross examination of the resected specimen showed a papilliferous tumor 4.5 × 3.5 × 2.0 cm in size. It appeared to consist of a mass of polypoid lesions that involved the intraluminal aspect of the duodenum. The cut surface revealed that it was non-capsulated and that the papillary lesion extended to the surface of the duodenal mucosa and involved the common bile duct (Figure 1). Light microscopy showed irregular tubules consisting of a single layer of tall columnar epithelial cells with elliptical nuclei. There were occasional goblet cells. The epithelium showed no nuclear stratification, but hyperchromatism and mitotic activity were occasionally observed. Small cyst formation presumably resulted from dilatation of glands secondary to obstruction of the ducts in the superficial layers. There was no evidence of invasion and no lymph node metastases. The pancreatic tissue was partly autolysed and showed features of chronic pancreatitis and papillary hyperplasia. Dysplasia of the bile duct was not observed.

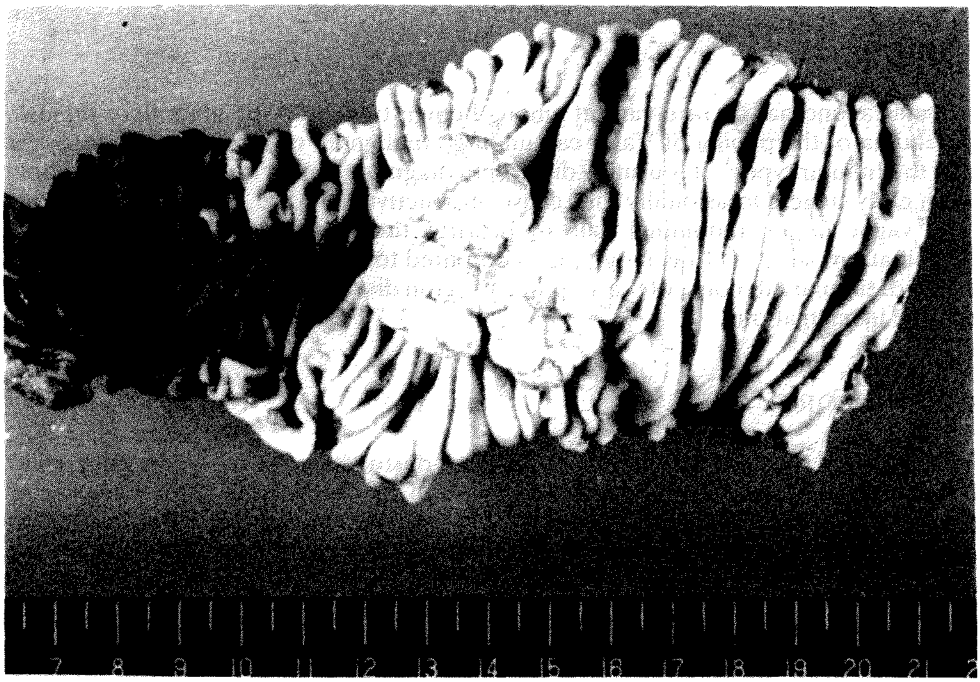

**Figure 1** The cut surface of the tumor was polypoid and developed from the ampullary region. The tumor extended to the duodenum and involved the common bile duct.

### DISCUSSION

In spite of numerous reports of carcinomas[4], benign tumors of the ampullary region are infrequent in incidence. Standard methods for their clinical diagnosis are not yet established. The commonest symptoms of ampullary tumor, apart from jaundice, are upper abdominal discomfort, nausea, and vomiting [5]. Although non-specific, these symptoms should trigger appropriate investigations.

Nakao [6] reviewed 538 cases of tumors of the ampullary region and reported that histological diagnosis was made at laparotomy in 85.5%, at autopsy in 10.8%, and by endoscopic biopsy in the remaining few. As yet there are no specific features of ampullary tumor on ultrasonography or CT scanning. Sampling by biopsy under ERCP examination may be inadequate because of the possibility of missing foci of carcinoma within an adenoma[1,7,8], although duodenoscopy is an invaluable screening method for tumors of the ampullary region. Thus, benign tumors of the ampullary region are frequently treated surgically on the suspicion of carcinoma.

Among benign tumors in the ampullary region, adenomas are rare. Yamaguchi[9] reported some cases out of 114 tumors arising from the ampulla of Vater. Adenoma of the ampulla comprised only 0.003% of digestive surgery cases in one series[10]. Starling collected 50 cases up to 1982[11], and the same 40 cases were reported in Japan up to 1987.

Scully and colleagues[12] described villous adenoma of the ampullary region as a sessile, mucosal mass composed of long, irregular villi with thin cores of loose fibrovascular tissue covered by a thick layer of epithelium. In another series of 19 cases of villous tumor of the duodenum, 63% of patients harboured cancer within the tumor[13]. However, the histological features of the present case differed from this description since we observed no long villi. A case intermediate in characteristics between ours and that of Scully was termed tubulo-villous adenoma by Delikaris[14]. Among 26 cases of adenoma reported in Japan with details of histological type[15], 19 were tubulo-villous, 12 were papillary type, 4 were tubular and 1 was papillo-tubular. In tubulo-villous adenomas, the tumor was composed of both tubular and villous structures, covered by a layer of tall columnar epithelial cells and in some areas containing goblet cells or Paneth cells. The epithelium of the tumor showed dysplasia with nuclear stratification, hyperchromatism, increased mitotic activity and secondary cyst formation. These histological features seemed similar to those of our case, though we observed little nuclear stratification or mitotic activity.

Delpy[16] reviewed 77 cases of adenocarcinoma of the ampulla and classified them according to the surgical procedures used: 14 cases were treated by transduodenal local excision, 38 by excision involving the biliary tract, 13 by partial pancreaticoduodenectomy, 5 by resection plus sphincterotomy, 3 cases by resection alone, and in 4 cases the tumor was not resected. Thus many kinds of surgical approaches have been reported and could be applied on the treatment of adenoma. However, carcinomatous change among such adenomas is well described[2]. Reviewing 1,174 autopsies for pancreatic carcinoma, Kozuka and co-workers[17] showed that non-papillary hyperplasia had a definite potential for malignant transformation through a sequence of papillary hyperplasia to atypical hyperplasia to carcinoma. That suggests that radical treatment is the method of choice for adenoma of the ampulla and that there is no place for local excision, tumor resection or sphincteroplasty.

### *References*

1. Archie, J.P. and Murry, H.M. (1978) Benign polipoid adenoma of the ampulla of Vater. *Arch Surg.*, **113**, 180–181
2. Uchida, Y., Tomonari, K., Shibata, O., Hamada, T., Shirabe, J., Matsumoto, K. and Yokoyama, S. (1986) Carcinoma in adenoma of the papilla of Vater. *Japanese Journal of Surgery,* **16**, 371–376
3. Kozuka, S., Tsuboue, M., Yamaguchi, A. and Hachisuka, K. (1981) Adenomatous resudue in cancerous papilla of Vater. *Gut,* **22**, 1031–1034
4. Hayes, D.H., Bolton, J.S., Willis, G.W. and Bowen, J.C. (1987) Carcinoma of the ampulla of Vater. *Ann. Surg.,* **206**, 572–577
5. White, S.H., Nazarian, N.A. and Smith, M.A. (1981) Periampullary adenoma causing pancreatitis. *Br Med J.,* **283**, 525
6. Nakao, N.L., Siegel, R.J. and Gelb, A.M. (1982) Tumors of the ampulla of Vater: early diagnosis by intraampullary biopsy during endoscopic cannulation. *Gastroenterology,* **83**, 459–464
7. Sculter, M.F., Oyasu, R. and Beal, J.M. (1976) Villous adenoma of the duodenim. *Am J Surg.,* **132**, 90–96
8. Baczako, K., Buchler, M., Berger, H.J., Kirkpatrick, C.J. and Haferkamp, O. (1985) Morphogenesis and possible lesions of invasive carcinoma of the papilla of Vater. *Hum Pathol,* **16**, 305–310
9. Yamauchi, K. and Enjoji, M. (1987) Carcinoma of the ampulla of Vater. *Cancer,* **59**, 506–515
10. Gmelin, E. and Weiss, H.D. (1981) Tumors in the region of the papilla of Vater. Diagnosis via endoscopy, biopsy, brush cytology, ERCP and CT-scan. *Eur J Radiol,* **1**, 301–306
11. Starling, J.R. and Turner, J.H. (1982) Villous adenoma involving the ampulla of Vater. *Amer Surgeons,* **48**, 188–190
12. Scully, R.E., Mark, E.J. and McNeely, B.U. (1984) Case records of the Massachusetts General Hospital: Weekly clinicopathological exercises. Case 30–1984. *N Engl J Med.,* **311**, 244–251
13. Rayn, D.P. and Schapiro, R.H. and Warshaw, A.L. (1986) Villous tumors of the duodenum. *Ann. Surg.* **203**, 301–306
14. Delikaris, P., Knudsen, F., Hjorth, L. and Balslev, I. (1983) Tubullo-villous adenoma of the papilla of Vater: a case with malignant changes, managed with local resection. *Mount Sinai J Med.,* **50**, 81–84
15. Delpy, J.C., Brunetom, J.N., Aubanel, D. and Lecomte, P (1983) Vaterian duodenal adenomas. *Forschr Rötgenstr,* **138**, 623–625
16. Sakato, M., Shimakura, K., Nozawa, K., Akamatsu, T., Nakama, H., Nakamura, Y., Matsuda, Y., Furuta, S. and Ueno, K. (1987) Five cases of adenoma of the papilla vater. *Gastroenterological Endoscopy,* **29**, 2506–2513 (In Japanese)
17. Kozuka, S., Sassa, R., Taki, T., Matsumoto, K., Nagasawa, S., Saga, S., Hasegawa, K. and Takeuchi, M. (1979) Relation of pancreatic duct hyperplasiato carcinoma. *Cancer,* **43**, 1418–1423

*Accepted by S. Bengmark on 15 November 1988*

### INVITED COMMENTARY

Although Drs Numata and Naguchi describe this tumour as a "papillotubular adenoma of the ampulla", I have a sneaking suspicion that it is in fact a tubulovillous adenoma of the duodenum. The intimate relationship of the terminal bile duct and pancreatic duct to the duodenal wall frequently presents difficulty in confidently ascribing tumours in this region to their precise tissue of origin. Klöppel and Fitzgerald subdivide such lesions into intraampullary and periampullary types but hedge their bets by including a third or mixed type[1]. Intraampullary tumours presumably arise from bile-duct or pancreatic-duct mucosa, whereas periampullary tumours (like the present case) arise from duodenal mucosa clothing the intestinal aspect of the papilla and protrude into the lumen of the bowel.

The authors mention four different histological types of adenoma in this region: tubulovillous, papillary, tubular and papillotubular. They state that the histological features of their own case resembled those of the commonest type, namely tubulovillous, though elsewhere in the report they explain that villi were not prominent. If we accept an origin from duodenal mucosa, then the tumour can be regarded as a variant (tubulovillous) of villous adenoma of the duodenum, a well-described lesion with a distinct propensity for carcinomatous change.[2–4] In one series of enteric (small-bowel) adenomas, 40 per cent were villous and no less than 65 per cent contained foci of cancer.[5] Likewise in the large intestine villous adenomas have a high malignant potential (41 per cent), whereas tubular adenomas do not (5 per cent); for tubulovillous adenomas, which contain elements of both histological patterns, the malignant potential is intermediate (22.5 per cent).[6]

Within the small intestine adenoma and adenocarcinoma have a strong predilection for the duodenum and within the duodenum for the descending portion and particularly the periampullary region.[7] Thus neoplasms such as this one, which could be described as papillary tumours of the papilla, are not particularly uncommon. One recent report of villous adenoma of the ampulla suggests the contrary, but since (like the present case) the lesion concerned is described as lying "around the papilla" the rarity seems spurious.[8] Within the small intestine some 40 per cent of carcinomas arise from the duodenum,[4,7] but within the extrahepatic biliary tree only 12 per cent arise from the ampulla[9], so that biliary excretion of carcinogens may or may not be relevant in the aetiology of periampullary cancer.

The adenoma-carcinoma sequence seems to pertain both to the instestinal tract (large and small intestine[5,6]) and to the biliary tract (gallbladder[10] and probably ampulla). Apart from the occasional endocrine tumour, adenoma of one or other histological subset is the only benign tumour to arise inside or around the ampulla with any degree of frequency. It is clearly sensible to regard every adenoma in this region as a potential carcinoma and to insist on complete excision. Although resection should ordinarily comprise pancreatoduodenectomy, as the authors recommend, it has to be admitted that in certain series the 5-year survival rate after local excision of ampullary carcinoma can equal or exceed that of pancreatoduodenectomy because of the perioperative mortality rate of the more major procedure.[11] If malignancy could definitely be excluded, local transduodenal resection would seem appropriate. Nevertheless I would have treated this particular tumour exactly as the authors describe, with the sole exception that I would prefer pylorus-preserving proximal pancreatoduodenectomy (PPPP) since carcinoma of the duodenal bulb is extraordinarily rare.[7]

R.C.N. Williamson
Hammersmith Hospital, UK

*References*

1. Klöppel, G. and Fitzgerald, P.J. (1986) Pathology of nonendocrine pancreatic tumors. In *The Exocine Pancreas. Biology, Pathobiology and Disease*, eds. Go, VLW, Gardner, J.D., Brooks, F.P., Lebenthal, E., DiMango, E.P., Scheele, G.A. Raven, New York 649–674
2. Batra, S.K., Schuman, B.M. and Reddy, R.R. (1983) The endoscopic variety of duodenal villous adenoma – an experience with ten cases. *Endoscopy* **15:** 89–92
3. Haglund, U., Fork, F-T., Genell, S., and Rehnberg, O. (1985) Villous adenomas in the duodenum. *Br. J. Surg.* **72,** 26–27
4. Williamson, R.C.N., Welch, C.E. and Malt, R.A. (1983) Adenocarcinoma and lymphoma of the small intestine. Distribution and etiologic associations. *Ann. Surg.*, **197**, 172–178
5. Perzin, K.H. and Bridge, M.F. (1981) Adenomas of the small intestine: a clinicopathologic review of 51 cases and a study of their relationship to carcinoma. *Cancer* **48**, 799–819
6. Morson, B.C. (1974) The Polyp-cancer sequence in the large bowel. *Proc. Roy. Soc. Med.* **67**, 451–457
7. Cooper, M.J. and Williamson, R.C.N. (1985) Enteric adenoma and adenocarcinoma. *World J. Surg.* **9**, 914–920
8. Malik, A.K., Baruah, M.K., Pravin, N. and Kataria, R.N. (1985) Villous adenoma of the ampulla of Vater. *Postgrad. Med. J.* **61**, 1077–1078

9. Anderson, J.B., Cooper, M.J. and Williamson, R.C.N. (1985) Adenocarcinoma of the extrahepatic biliary tree. *Ann. Roy. Coll. Surg. Engl.* **67**, 139–143
10. Williamson, R.C.N. (1988) Acalculous disease of the gall bladder. *Gut* **29**, 860–872
11. Knox, R.A. and Kingston, R.D. (1986) Carcinoma of the ampulla of Vater. *Br. J. Surg.* **73**, 72–73
12. Cooper, M.J. and Williamson, R.C.N. (1985) Conservative pancreatectomy. *Br. J. Surg.* **72**, 801–803

### INVITED COMMENTARY

Since the initial report of Golden in 1928 of benign tumours of the duodenum many case-reports of these "rare" tumors have been published[1]. In the present study a patient with a papillotubular adenoma was treated by pancreaticoduodenectomy. The authors conclude that adenoma in the ampullary region should be treated as adenocarcinoma and there is no place for local excision, tumor resection and sphincteroplasty.

Controversy in literature however exists concerning the therapeutical approach of patients with villous tumors of the duodenum. The following factors should be taken into consideration for the management of these lesions.

1. Are these adenomas indeed premalignant and is transition of adenoma to carcinoma proven?

A review of the Cleveland Clinics reported malignancy in these villous tumors in 47% and some of these were carcinoma in situ[2]. In another series even in 12 of 19 patients (69%) the villous tumor had "malignant elements"[3]. A survey of the literature of tumors of the small intestine demonstrated a relationship between the frequency of adenomas and carcinomas. Adenomas and carcinomas were respectively found in 104 and 735 patients in the ampullary region; in 94 and 180 patients in the remaining part of the duodenum; and in 20 and 72 patients in the jejunum. Regarding the age and sex distribution an adenoma-carcinoma sequence was suggested[4]. The increased risk of upper gastrointestinal carcinoma in the polyposis coli patient (most frequently duodenum or periampullary region) also supports an adenoma-carcinoma sequence[5]. One of our patients developed four years after removal of a papillary lesion of the ampulla a papillary adenocarcinoma which also strongly suggests the transition into carcinoma[6].

2. Is endoscopy with biopsy sufficient to prove the diagnosis or more important, to exclude malignancy?

The areas of malignant changes are frequently focal and superficial biopsies cannot exclude malignancy[7,8]. In one series with more than 50% of the patients suffering from invasive carcinoma the biopsy preoperatively showed only villous adenoma[3]. Therefore, the entire specimen should always be removed for examination. This does not exclude endoscopic extraction as a possible treatment modality for some small lesions[8]. The use of endoscopic ultrasonography in carcinoma of the papilla of Vater has been described recently and this technique could also be promising in determining the extent of adenomas of the duodenum, thus selecting patients for local treatment[9].

3. Is the recurrence rate high and is a malignancy in a recurrent lesion common?

Recurrence rates have been reported between 20–60% depending on the type of surgery used. Recurrence has even been described 10 years after removal of the lesion [10]. However, malignancy in recurrent tumors, as in our patient is not common.[6].

Considering the above mentioned factors there are 4 different treatment modalities: radical surgery; local surgical excision; endoscopic removal and laser treatment. In the present report radical surgery has been suggested. This preference for pancreaticoduodenectomy, presumably resulting in better local control, is stated by many others especially in younger patients[3,6]. Although mortality of this procedure is currently acceptable morbidity figures remain between 30–60%. On the other hand, extended local excision and if necessary, reconstruction of the distal bile and pancreatic ducts, as reported by Krukowski et al and van der Heyde, also showed good results and limited recurrence[11,12]. Long term follow-up with endoscopy is mandatory however because recurrence can be expected even after 10 years[10]. Endoscopic removal can be considered for the small pendulated tumors if the entire lesion can be removed[7]. In one patient we performed laser treatment for a recurrent lesion. Recently this technique was also used primary treatment[13]. Complete destruction was obtained in 7 of 8 patients (follow-up 14–53 month) and one patient had a recurrence after 2 years[13]. The role of the laser as primary treatment needs further evaluation.

Regarding the different treatment modalities the strategy of treatment as suggested by Galandiuk (local excision for benign lesions or superficial invasion and radical resection for invasive adenocarcinoma) seems well balanced[2].

Dirk J. Gouma

Rijksuniversiteit Limburg

### *References*

1. Golden, R. Non-malignant tumors of the duodenum. *American J. of Rontgenology, Radiation Therapy, and Nuclear Medicine 1928;* **20**, 405
2. Galandiuk, S., Hermann, R.E. and Jagelman, D.G. *et al.* (1988) Villous tumors of the duodenum. *Ann Surg.*, **207**, 234–239
3. Ryan, D.P., Schapiro, R.H. and Warshaw, A.L. (1986) Villous tumors of the duodenum. *Ann Surg.*, **203**, 301–306
4. Sellner, F. (1987) Hypothesis on the existence of an adenoma-carcinoma sequence in the small intestinal. *Z-Gastroenterol,* **25**(3), 151–165
5. Jagelman, D.G., DeCosse, J.J. and Bussey, H.J.R. (1988) Upper gastrointestinal cancer in familial adenomatous polyposis. *The Lancet,* **21**, 1149–1150
6. Gouma, D.J., Obertop, H. and Vismans, J. et al. (1987) Progression of a benign epithelial ampullary tumor to adenocarcinoma. *Surgery,* **101**, 501–503
7. Perzin, K.H. and Bridge, M.F. (1981) Adenomas of the small intestine: a clinical review of 51 cases and a study of their relationship to carcinoma. *Cancer,* **48**, 799–819
8. Sivak, M.V. (1988) Clinical and endoscopic aspects of tumors of the ampulla of Vater. *Endoscopy,* **20**, 211–217
9. Yasuda, K., Mukai, E. and Cho E. et al. (1988) The use of endoscopic ultrasonography in the diagnosis and staging of carcinoma of the papilla of Vater. *Endoscopy,* **20**, 218–223
10. Muir, I.M., Ballantyne, K.C. and Wilkins, J.L. Tubulovillous neoplasms of the duodenum. *Br. J. Surg.*, **75**, 829
11. Krukowski, Z.H., Ewen, S.W.B. and Davidson, A.I. et al. (1988) Operative management of tubulovillous neoplasms of the duodenum and ampulla. *Br. J. Surg.*, **75**, 150–153
12. Eggink, W.F., van Berge Henegouwen, G.P. and van der Heyde, M.N. et al. (1988) Tumors of the ampulla of Vater treated by local resection: a report of five cases. *The Netherlands J. of Surg.*, **40**, 110–114
13. Lambert, R., Ponchon, T. and Chavaillon, A. *et al.* (1988) Laser treatment of tumors of the papilla of Vater. *Endoscopy,* **20**, 227–232

